# Early-Onset Invasive Pulmonary Aspergillosis in a Liver Transplant Patient: A Case Report

**DOI:** 10.7759/cureus.42554

**Published:** 2023-07-27

**Authors:** Juan P Espinosa-Leon, Theresa Feng, Andres de Lima, Brian P O'Gara

**Affiliations:** 1 Anesthesiology, Critical Care and Pain Medicine, Beth Israel Deaconess Medical Center, Harvard Medical School, Boston, USA

**Keywords:** invasive fungal infection, liver transplantation, invasive pulmonary aspergillosis, id critical care, clinical case report, antifungal agents

## Abstract

Invasive pulmonary aspergillosis (IPA) in liver transplant patients remains rare but exceedingly fatal. The diagnostic challenges associated with this condition are compounded by its infrequent onset within the first two weeks following transplantation. Moreover, therapeutic management is complex due to the intricate drug interactions between triazole antifungals and calcineurin inhibitor immunosuppressants. We present the case of a 63-year-old male who underwent uncomplicated liver transplantation (LT) and developed early-onset IPA. Despite maximal efforts, the patient expired. This report aims to underscore the vital importance of timely diagnosis and therapy in preventing the insidious progression of invasive disease and subsequent mortality.

## Introduction

Liver transplantation (LT) recipients face an elevated risk of infections due to underlying hepatic impairment and immunosuppression during the early post-transplant period [1,2]. Invasive fungal infections pose significant morbidity and mortality risks. Although rare in this population, invasive pulmonary aspergillosis (IPA) has mortality rates as high as 85% [1-5]. Early detection and effective management are crucial to improve outcomes and reduce mortality. Nonetheless, diagnosing IPA in LT patients presents significant challenges.

We aim to address the unique obstacles faced in diagnosing and managing IPA in LT recipients, specifically focusing on a case of early-onset IPA. This report emphasizes the need for improved pre-transplantation screening, early suspicion of IPA, and enhanced diagnostic approaches to detect and manage the disease, enhancing outcomes for LT recipients.

The patient's next of kin has provided written HIPAA authorization to share this case and images for publication. We adhered to the CARE guidelines.

This article was previously presented as a meeting abstract at the SOCCA & IARS Annual Meetings on April 14 and 16, 2023, in Denver, CO.

## Case presentation

A 63-year-old male with a past medical history of hypertension, coronary artery disease status post percutaneous coronary intervention, obstructive sleep apnea on continuous positive airway pressure, history of COVID pneumonia, type 2 diabetes, and non-alcoholic steatohepatitis cirrhosis complicated by esophageal varices was admitted to the hospital for deceased donor LT. The patient's intraoperative course was uncomplicated. He was extubated and admitted to the ICU for postoperative care. Per institutional guidelines, his immunosuppression regimen consisted of mycophenolate mofetil, tacrolimus, and steroids. For antimicrobial prophylaxis, he received empiric fluconazole, aztreonam, metronidazole, and trimethoprim-sulfamethoxazole. The perioperative course was complicated by acute kidney injury, necessitating initiation of hemodialysis on postoperative day (POD) two.

On POD four, the patient started complaining of dyspnea, frequent hiccups, and coughing spells associated with episodes of transient hypoxia. A chest X-ray revealed right middle and lower lobe opacities concerning aspiration pneumonia. Broad-spectrum empiric antibiotics (vancomycin and cefepime) were started. Three days later, the patient reported dizziness and shortness of breath. Clinical examination revealed diaphoresis, tachycardia, hypotension, and signs of respiratory distress. An increased amount of bloody drainage was noted from the intraabdominal drain. Laboratory findings were significant for leukocytosis (29.6 cells/uL) and anemia (hemoglobin level of 6.8 g/dL), for which he received one unit of packed red blood cells. An abdominal CT revealed a 5 x 3 cm posterior perihepatic hematoma. The patient was readmitted to the ICU for close hemodynamic and respiratory monitoring.

**Figure 1:**
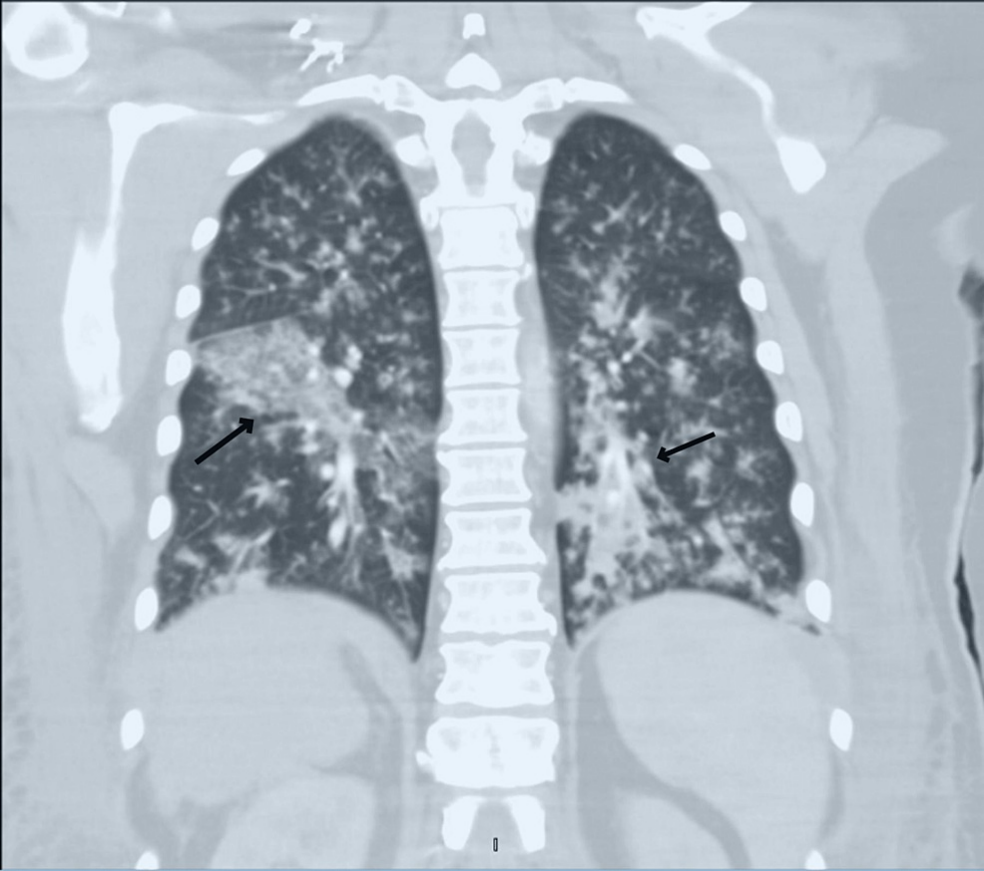
Chest CT Chest CT obtained on POD nine demonstrates consolidative opacities (arrows) throughout both lungs and moderate, diffuse bronchial wall thickening with scattered endobronchial secretions consistent with widespread multifocal pneumonia and bronchopneumonia

**Figure 2:**
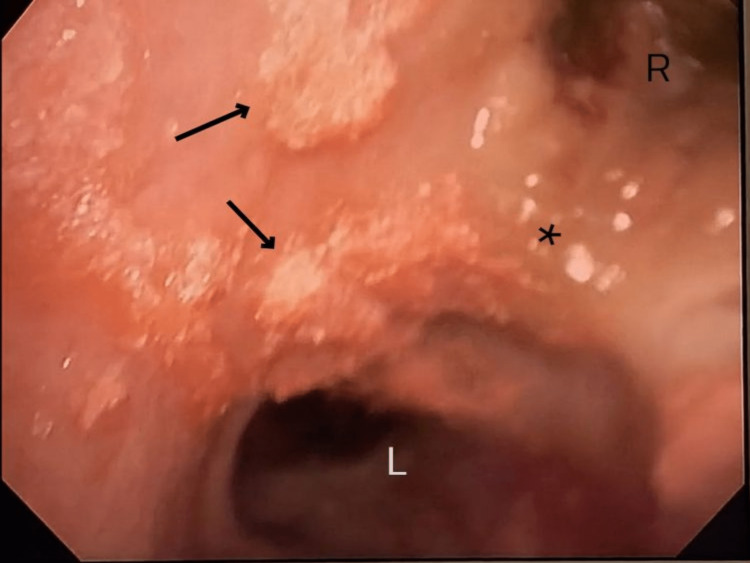
Right-mainstem bronchoscopy The image obtained on POD nine demonstrates a screen capture from diagnostic flexible bronchoscopy at the right-mainstem bronchus. Findings were consistent with general inflammation with erythema, nodularity, and scattered pseudomembranous lesions (arrows) with copious thick purulent secretions in proximal airways, suggestive of bronchopneumonia. *, carina; L, left mainstem bronchus; R, right mainstem bronchus

On the ninth POD, the patient's tachypnea and hypoxemia worsened, prompting endotracheal intubation. A subsequent chest CT (Figure 1) revealed bilateral consolidative opacities with diffuse bronchial wall thickening, consistent with bronchopneumonia and multifocal pneumonia. Bronchoscopy (Figure 2) demonstrated diffuse nodularity and scattered pseudomembranous lesions with copious thick purulent secretions and significant erythema of the bronchial epithelium. Endobronchial biopsies and bronchoalveolar lavage were performed, with biopsies revealing necrosis and rare hyphae. Based on the progressive clinical deterioration and the findings on imaging and histopathology, there was a concern for invasive pulmonary fungal infection; thus, amphotericin B was initiated. *Aspergillus fumigatus* was subsequently isolated from respiratory cultures. Over the next five days, the patient's clinical condition deteriorated significantly, requiring rapidly escalating vasopressor support and the addition of voriconazole, with the progression of septic shock and multiorgan failure notable for ongoing kidney failure, profound coagulopathy, and severe mixed metabolic and respiratory acidosis. Following the goals of care discussion with family members, a decision was made to transition care to comfort-focused measures. The patient expired on POD 15. Post-mortem lung tissue cultures were notable for *Aspergillus fumigatus*.

## Discussion

IPA is a rare but highly lethal disease in solid organ transplant recipients (SOTr). While clinical outcomes for SOTr patients with IPA have improved since the early 2000s, LT patients continue to experience poor outcomes [2]. Incidence varies widely, ranging from 1.2% to 5.8%, with significant variability within this population [1-3,6]. Despite the availability of antifungal therapy, mortality rates for IPA in LT have been reported as high as 85%, compared to 15% in other SOTr populations [2].

In the case of suspected IPA, a high index of clinical suspicion and a meticulous diagnostic approach combining laboratory, radiology, and histopathology methods are crucial. However, the diagnostic tools currently available for IPA in LT patients may delay diagnosis and treatment, contributing to disseminated disease and high mortality [2]. The most commonly used enzyme immunoassays, galactomannan (GM) and beta-D-glucan (BG), have low diagnostic accuracy in LT patients [2,7,8]. A meta-analysis by Pfeiffer et al. reported a sensitivity of 22% and specificity of 84% for GM in solid SOTr, compared to 82% and 86% in hematopoietic stem cell transplant patients and 70% and 92% in patients with hematologic malignancies [8].

External factors may alter the accuracy of these tests, and caution must be exercised when diagnosing IPA based on antigen testing. In a randomized clinical trial of high-risk LT patients, 45.5% of patients without IPA had a positive baseline GM, indicating a high false positivity rate that may be increased by external factors commonly encountered during the clinical course of LT patients, making these tests poor aids for decision making. Beta-lactam antibiotics, infusion with Plasma-Lyte solution, use of sterile gauze and other equipment during surgery, biofilms from central venous catheters, renal replacement therapy (RRT), and even intestinal bacterial translocation can confound the specificity of the test, while exposure to antifungals decreases GM's sensitivity [7]. Our patient had a positive BG and GM six months before transplantation (04/2022) without clinical signs or symptoms of a fungal infection. It was difficult to determine whether this was a false positive test or if the patient was colonized with *Aspergillus* before surgery. The latter could explain the rapid onset of disease after transplant, as previously hypothesized [9].

Imaging findings, while not pathognomonic for IPA, can be helpful in its diagnosis. A retrospective analysis of 2,150 LT patients' chest radiographs and CT scans revealed that nodules <3 cm (64%), masses >3 cm (36%), and patchy consolidations (20%) were the most common radiological findings in those with IPA [6]. In this case, a normal chest X-ray before surgery was followed by right basal opacification on POD two, initially thought to be atelectasis. On POD six, a CT scan revealed patchy multifocal opacities compatible with pneumonia. Although it cannot be confirmed, these early radiological findings may have been the first indication of IPA, even before presenting severe symptoms.

LT recipients are at a heightened risk for IPA due to multiple host and surgical factors. Risk factors include a model for end-stage liver disease (MELD) score greater than 20, bacterial or cytomegalovirus infection within the first month after transplantation, renal insufficiency, re-transplantation, cytomegalovirus donor-positive recipient-negative serology, bilirubin levels greater than 1.5 mg/dL at the time of LT, prolonged ICU stay, RRT following LT, and a positive *Aspergillus* serum antigen [1,2,10]. According to Fortun et al., the requirement of RRT after LT, re-transplantation, and a positive *Aspergillus* serum antigen antigenemia are independently associated with developing IPA [1]. In the case of our patient, the RRT requirement was the only identified risk factor by the time they developed symptoms.

According to the latest guidelines from the Infectious Diseases Society of America, there is a lack of evidence addressing specific prophylactic therapy against *Aspergillus* in nonlung solid SOTr. Thus, recommendations are based on the institutional epidemiology of infection and the patient’s individual risk factors [11]. Nonetheless, prophylaxis with voriconazole is effective in preventing IPA in high-risk liver LT recipients, and recommendations exist for patients with specific individual risk factors. These include a MELD score of >25 or those meeting two or more of the following criteria: pretransplant ICU length of stay of >24 hours, pretransplant respiratory failure requiring mechanical ventilation, inotropic therapy requirements, need for RRT, re-transplantation, fulminant hepatic failure, and combined kidney-LT [12].

Voriconazole is the first-line treatment for IPA [13]. Like other triazoles, it inhibits the cytochrome P450 (CYP450) 3A4 isoenzyme responsible for metabolizing immunosuppressants. Caution must be exercised in LT patients receiving tacrolimus and other calcineurin inhibitors to prevent increased blood levels and drug toxicity, which may require dose reductions and monitoring of drug concentration in blood [5,14]. We anticipated the need for long-term antifungal treatment in conjunction with tacrolimus; therefore, we administered amphotericin B rather than voriconazole.

## Conclusions

The development of IPA in a liver transplant recipient without significant risk factors is uncommon. Prophylaxis with voriconazole is effective in preventing IPA in high-risk LT recipients. However, efforts should be made toward pre-transplantation screening for *Aspergillus*, early suspicion of the disease, and an improved diagnostic strategy to detect and manage the disease promptly.
